# Botulinum Toxin Type A Injections in the Bladder Wall—An Effective Treatment for Urinary Incontinence with Low Long-Term Adherence

**DOI:** 10.3390/toxins18040170

**Published:** 2026-04-01

**Authors:** Francisco Cruz, Martin C. Michel, Yasuhiko Igawa

**Affiliations:** 1 Department of Urology, Faculty of Medicine, University of Porto, 4200-319 Porto, Portugal; 2 Department of Urology, Hospital São João, 4200-319 Porto, Portugal; 3 RISE-Health Institute for Research, 4200-319 Porto, Portugal; 4 Department of Pharmacology, University Medical Center, Johannes Gutenberg University, 55131 Mainz, Germany; marmiche@uni-mainz.de; 5 Pharmaceutical Medicine, Charlotte Fresenius University, 65185 Wiesbaden, Germany; 6 Department of Urology, Nagano Prefectural Shinshu Medical Center, 1332 Suzaka, Nagano 382-8577, Japan; yxigawa@gmail.com

**Keywords:** botulinum toxin, urinary incontinence, neurogenic detrusor overactivity, overactive bladder, bladder administration

## Abstract

Botulinum toxin type A (BoNT/A) injection into the bladder wall is a milestone in the treatment of urinary incontinence in patients with neurogenic detrusor overactivity (NDOi) or overactive bladder syndrome (OABi) who are refractory to or unable to tolerate oral or transdermal therapies. However, the efficacy of BoNT/A is hampered by the low long-term adherence of patients to a treatment that requires repeated bladder injections under cystoscopy control. The discontinuation is particularly evident among incontinent patients with spontaneous voluntary voiding, regardless of whether the cause is NDOi or OABi, although clearly more marked among the latter group. In addition to the bother and pain associated with repeated cystoscopies, these patients show low tolerance to the high incidence of urinary tract infections (UTIs) and transient urinary retention, the two most common adverse events. Fewer injection points may render treatments less painful, apparently without reducing efficacy, but will not avoid the need for repeated cystoscopies, and no studies have demonstrated that such modification increases adherence. Eventually, accessing the bladder wall for BoNT/A administration via a transabdominal approach, under real-time ultrasound guidance, may overcome trans-urethral limitations, but the technique’s reproducibility remains unknown. An intensive investigation is ongoing to identify aids that facilitate the passage of the large, fragile BoNT/A molecule across the urothelium to reach the bladder nerves without injections. Electromotive Drug Administration (EMDA) of BoNT/A demonstrated efficacy and safety over a 6-year follow-up in NDOi patients at a single center, but the results were not reproduced at other institutions. The application of shock waves to the bladder using shock waves generated by Extracorporeal Shock Wave Lithotripsy (ESWL) machines to tear the urothelium and facilitate the passage of BoNT/A instilled in the bladder is ingenious, but the experience is very limited. Dimethyl sulfoxide, liposomes, and thermal-reversal hydrogel to deliver the toxin failed in pilot trials. BoNT/A in nano-formulations has high heat stability, resistance to pH changes, and to enzymatic degradation. Extended efficacy in dermal and intramuscular pilot applications is promising but needs to be replicated in the bladder.

## 1. Introduction

One of the major advances in the management of urinary incontinence associated with neurogenic detrusor overactivity (NDOi) or overactive bladder syndrome (OABi) is the introduction of intravesical injections of botulinum toxin subtype A (BoNT/A) [1,2,3]. The potency of BoNT/A brands is not interchangeable. This led to the introduction of the non-proprietary names onabotulinum toxin A (OnabotA), abobotulinum toxin A (AbobotA), and incobotulinum toxin A (IncobotA) for Botox^®^, Dysport^®^, and Xeomin^®^, respectively [4,5]. Although BoNT/A is available in more than 40 botulinum toxin products, only these brands have a robust reputation in the treatment of urinary incontinence associated with NDO (NDOi) and OAB (OABi).

BoNT/A is more effective than oral anticholinergic medication to control NDOi [6], and oral or transdermal anticholinergic medication or oral mirabegron to control OABi [7]. In pivotal trials with NDOi patients, more than 60% of the participants reported clinically relevant improvement or cure after the first treatment [8,9,10,11], a proportion that may further increase in subsequent treatments [12]. Regarding OABi patients, pivotal trials reported that at least 60% had a marked or complete (cure) improvement [13,14]. However, the duration of BoNT/A effect injected in the bladder, although long, is limited in time, requiring repeated applications under cystoscopy control at intervals ranging from less than 6 months up to more than 12 months [15,16,17]. In addition, BoNT/A injections are associated with a high incidence of mild to moderate adverse events, mainly lower urinary tract infections (UTIs) and transient urinary retention among patients with spontaneous voiding, requiring variable periods of (self-) catheterization [8,9,10,11,13,14]. Altogether, failure to achieve the desired improvement, the bother and pain caused by repeated cystoscopies, and the risk of adverse events may deceive many patients, leading to a premature abandonment of BoNT/A treatment. The pivotal clinical trials could not sort out this negative side of long-term BoNT/A treatment, which only became evident when real-world cohorts were reported. This narrative review first summarizes the classical method of intravesical injections, the rate of discontinuation, and the variations to BoNT/A bladder injections that have been explored to facilitate bladder administration. Then, the techniques under investigation to substitute intravesical injections will be reviewed. A systematic review was not considered due to the low number of papers stating discontinuation rates and the absence of comparisons between methods. Moreover, the reports used different time points for analysis, and the reasons for stopping treatment were described in distinct forms.

## 2. In the Real World, Adherence of Patients to Long-Term BoNT/A Treatment Using the Approved Methods of Administration Is Lower than Expected from Pivotal Trials

In real-world clinical practice, discontinuation of BoNT/A treatment delivered via cystoscopy is a concern for the dissemination of this highly effective treatment for urinary incontinence. Regardless of whether BoNT/A is injected under flexible cystoscopy control and lidocaine anesthesia, or under more expensive spinal or general anesthesia to further reduce the discomfort, the discontinuation rate is very high [18].

The method of BoNT/A administration approved by the Food and Drug Administration (FDA) and the European Medicines Agency (EMA) for NDOi derives from the pivotal studies with OnabotA [8,9] or with AbobotA [10]. OnabotA 200 U diluted in 30 mL of saline is distributed by 30 injections of 1 mL above the trigone. For AbobotA, 600 U or 800 U are dissolved in 15 mL of saline, and injections of 0.5 mL are distributed at 30 points above the trigone. In both cases, injections are carried out under cystoscopy control. Most adverse events were observed in non-self-catheterizing MS patients, 60% of whom developed UTIs, and 30% had to start catheterization to empty the bladder [8,9]. Decreasing the OnabotA dose from 200 U to 100 U in MS patients with a post-void residual volume at baseline less than 150 mL halved the proportion of patients with UTIs and urinary retention but did not eliminate these complications [11]. Concerning OABi, the FDA and EMA only approved OnabotA 100 U, the only toxin tested in pivotal trials. It should be injected in 20 points above the trigone, each receiving 0.5 mL of the toxin solution [13,14]. FDA and EMA have not yet approved AbobotA, IncobotA, or other brands for OABi. Discontinuation rates in the NDOi and OABi pivotal trials were very low, around 10%. However, the trials were not designed to investigate adherence to the treatment. Most patients received only 1–2 treatments during the main phase of the trials. The long-term extensions in which patients were invited to remain on treatment for several years were designed to investigate efficacy and adverse events along multiple treatment cycles, but not real-world adherence [15,17].

The discontinuation rates, reasons for discontinuation, and factors associated with discontinuation with the standard method of intravesical BoNT/A injection for NDOi and OABi patients are summarized in Table 1. Long-term persistence with a treatment is influenced by patient expectations. How much incontinence disrupts daily activities, the type of bladder dysfunction, and caregiver and institutional backing to overcome the fatigue typically associated with chronic treatments play important roles. NDOi patients of spinal origin, particularly those followed in specialized institutions, receive a high level of information about the potential life-threatening consequences of incorrect management of the bladder dysfunction. For this group of patients, persistence on BoNT/A treatment is not solely motivated by the stigma of incontinence. According to real-world cohorts, NDOi patients discontinue treatment due to an insufficient improvement of urinary incontinence and to the natural progression of underlying neurological disease, rather than to the potential bother or pain caused by the repeated treatments. In a large cohort of spinal cord-injured patients treated with AbobotA 500–1000 U over 8 years, the discontinuation rate was 11% due to insufficient response and 1.5% due to the bother caused by repeated treatments [16]. In another large cohort of NDOi patients of mixed etiologies treated with OnabotA or AbobotA, the 10-year discontinuation-free rate was higher, 49.1%, most probably reflecting the different patient selection. Nevertheless, the most common reason for discontinuation was also unsatisfactory results. Other reasons were the patient’s decision in 28.1%, non-botulinum toxin A-related improvement of urinary incontinence in 14.1%, and progression of the neurological condition in 12.5%. Adverse events were the cause in only 1.6% of the patients [19]. Breaking down by etiology, discontinuation rate was higher among patients with spina bifida than among patients with spinal cord injury (SCI), which is likely to be related to the poorer bladder properties of spina bifida patients [19]. In NDOi patients of Multiple Sclerosis (MS) etiology, adherence is substantially lower. In a report from the national French database of MS patients, among those who initiated BoNT/A therapy in 2016, only 47% received at least six BoNT/A injections during a period of six years (analysis until 2022) [20], the minimum number of treatments expected from the estimated duration of the effect of OnabotA in NDOi patients enrolled in pivotal trials [8,9,10,16]. The reasons why more than half of the patients received fewer than six injections were related to poor efficacy, severity of the underlying condition, and compliance with the treatment. In another MS cohort originating from 11 French hospitals, around 80% of the patients were still under BoNT/A treatment 5 years after the first injection. Discontinuation was associated with MS severity. Patients with a low Expanded Disability Status Scale (EDSS) score (<6) and a relapsing–remitting form had a 5.5% discontinuation rate. In contrast, patients with an EDSS ≥ 6 and a progressive type had a discontinuation rate 6 times higher, i.e., above 30% [21]. In this cohort, the main reasons for MS patients to abandon BoNT/A treatment were difficulty in doing self-catheterization and insufficient results [21]. From these series, one can conject that in the NDOi population, in addition to good caregiver support, a smart patient selection will play a key role in persistence. Caregivers should realize that BoNT/A injections do not correct low bladder capacity and poor compliance caused by intense bladder fibrosis. This might explain the poor adherence of spina bifida patients, disappointed by the poor continence gain with BoNT/A bladder injections. MS patients with an EDSS > 6–7 and a progressive disease may not also find a clear benefit from treatment. Most will be bedridden and will have poor manual dexterity. Limited mobility will decrease the drive to attend repeated appointments for BoNT/A administration, and reduced manual dexterity will make self-catheterization difficult if urinary retention develops.

In the pivotal randomized clinical trial with NDOi patients, antibiotic prophylaxis was used on the presumption that bacteriuria could reduce the efficacy of the toxin [8,9]. However, many of the injected SCI patients relied on bladder catheterization and presented chronic bacteriuria. Thus, the relevance of antibiotic prophylaxis before BoNT/A injection in SCI patients was investigated in 154 patients undergoing a total of 273 OnabotA treatments for refractory NDOi. Patients without clinical signs of UTI underwent injections without antibiotic prophylaxis. Bacteriuria was present in 73% (200/273) of all patients before treatment. Following treatment, symptomatic UTIs occurred in 7% (5/73) of cases with sterile urine culture and in 5% (9/200) with bacteriuria. These results suggest that routine antibiotic prophylaxis is not required before botulinum toxin injection in SCI patients with asymptomatic bacteriuria [22]. However, it is unlikely that the antibiotic protocol before injections might affect BoNT/A discontinuation rates.

OABi is nowadays the main indication for bladder injections of BoNT/A, surpassing NDOi by a large margin [20], despite the high discontinuation rate that overpasses the rates seen among neurogenic patients. OABi patients are not at risk of deterioration of the upper urinary tract. So, patients’ expectations related to the treatment will play a fundamental role in persistence on BoNT/A treatment. Not surprisingly, poor tolerability caused by the need for repeated treatments under cystoscopy control, followed by insufficient efficacy and the fear of adverse events, including UTIs and the occasional need for transient self-catheterization, are the main reasons for stopping treatment. Interestingly, these drawbacks had not been uncovered in the long-term extension of the pivotal phase III trials [23], but they became evident when real-world adherence to treatment started to be reported. Mohee and co-workers [24] found a discontinuation rate of 61.3% at 36 months and 63.8% at 60 months. In another cohort reported by Marcelissen and co-workers with at least 5 years of follow-up after the first injection, only 30% of the patients were still on OnabotA treatment at the last visit. Discontinuation occurred after the first (79%) or second (19%) treatment. The main reason cited by patients for discontinuation was related to tolerability (43%), while only 27% indicated insufficient efficacy [25]. In another study in which OABi patients received AbobotA up to 500U, the discontinuation rate at last follow-up was 88%. The main cause of discontinuation was primary failure (35%), followed by tolerability issues (20.3%). Improvement of the condition was the reason to stop treatment in 23.7% of the patients [26]. According to the national French database, only 31% of the OABi patients received more than two treatments, and only 19% received four or more injections between 2016 and 2022 [20]. In another study from a tertiary Portuguese Hospital, around 70% of the women either refused or did not attend the appointment for the second or the third treatment, despite the low rate of complications observed. Invasiveness of the treatment was the main reason for discontinuation. For those who requested a second treatment, the median interval was extremely long, around 18 months [27]. In male OABi patients, the discontinuation rate is also very high. In a total of 88 males with a mean follow-up of 6 years, only 25% continued BoNT-A treatment at the last follow-up. Insufficient effect and tolerability due to urinary retention, which obliged 24% of the males to use intermittent catheterization or indwelling catheters at some point during the follow-up [28]. 

Exceptions to the high rate of discontinuation in real-world reports are rare. One was described by a dedicated OAB clinic that maintains high interaction with the patients. The 6-year persistence rate on OnabotA treatment was 68%, a remarkably high rate in line with the high satisfaction reported by patients with the service offered by the institution. Patients who abandoned did so due to symptomatic improvement or returned to oral medication [29]. In another predominant OABi population treated in a medium-sized institution, the discontinuation rate at five years was 25%. Interestingly, the intervals between treatments were very long, around 1 year [30].

In conclusion, if BoNT/A treatment is to be expanded among OABi patients not responding or not tolerating oral medication, new methods of toxin administration to the bladder need to be implemented in the clinic.

**Table 1:** The discontinuation rates, reasons for discontinuation, and factors associated with discontinuation with standard intravesical BoNT/A injection for NDOi and OABi patients.

Patient Types	Type and Dose of BoNT/A	Evaluation Time Point	Discontinuation Rate	Reasons for Discontinuation	Factors Associated with Discontinuation	Ref.
NDOi due to SCI	AbobotA 500–1000 U	mean: 4 y (median: 52 mo; range: 16–91 mo)	11.6%	11.6% insufficient efficacy 1.5% the bother by repeated treatments		[14]
NDOi with mixed etiologies	OnabotA 200–300 U or AbobotA 500–1000 U	5 y 10 y	36.1% 50.9%	43.7% insufficient efficacy (failure) 28.1% patient decision 14.1% non-botulinum toxin A-related improvement of urinary incontinence 12.5% neurological condition progression	Higher discontinuation rate in Spina bifida than SCI or MS	[19]
NDOi due to MS	Not specified (probably all brands)	6 y	53%	Poor efficacy, severity of the underlying condition, and compliance		[20]
NDOi due to MS	OnabotA 100–300 U or AbobotA 500–1000 U	5 y	19%	46% Difficulty in doing self-catheterization 46% insufficient efficacy 8: Other reasons	MS severity	[21]
OABi 75.9% NDOi 24.1%	OnabotA 100–300 U	3 y 5 y	61.3% 63.8%	55.9% tolerability issues 44.1% insufficient efficacy (27.4% primary failure; 16.7% secondary failure)	Incontinence at baseline and younger age (≦50) increased abandonment	[24]
Females OABi	OnabotA	5 y	70%	43% tolerability issues 27% insufficient efficacy		[25]
OABi Female	OnabotA	15 y	70%	Tolerability		[27]
OABi male and female	All brands	6 y	69%	Lack of efficacy 36% Spontaneous improvement 24% Tolerability 20%		[20]
Females OABi	OnabotA 200 U	6 y	32%	Discontinuation due to symptomatic improvement or return to oral medication		[29]
OABi	AbobotA 150–500 U	5 y	88%	37.3% insufficient efficacy 23.7% persistent improvement of symptoms 20.3% tolerability issues	Male gender	[26]
Males OABi	OnabotA 100–300 U	mean 69 mo	75%	53.0% insufficient efficacy 40.9% tolerability issues		[28]
IDO andNDO	OnabotA 200 U for OAB 300 U for NDO	5 y	25%	17% insufficient efficacy 26% inadequate bladder emptying requiring Intermittent Self-Catheterization		[30]

## 3. Variations to the Approved Protocol for BoNT/A Injections Under Cystoscopy Control Did Not Investigate the Effect on Adherence to Treatment

Multiple variations to the approved form of BoNT/A administration to the bladder have been explored over the years, in small, short-term trials. Although the objective was to facilitate BoNT/A administration and decrease patient discomfort, none specifically defined long-term adherence as a key outcome. Some were investigated with the clear objective of increasing efficacy. Injections in the trigone were investigated because this is the area of the bladder with the highest content of sensory fibers [31]. In a small randomized trial, OABi patients receiving AbobotA 500 U, divided by 15 injections above the trigone plus 5 injections in the trigone, had superior symptomatic improvement than those who received all 20 injections above the trigone. The mean postvoid residual volume and the incidence of clean intermittent self-catheterization were similar in the two groups [32]. Another study compared detrusor, suburothelial, or trigonal injections of 100 U of OnabotA in OABi patients [33]. Detrusor injections had a more robust and lasting symptomatic improvement than suburothelial injections. Trigone-only injections were less effective and durable, although risk-free of causing urinary retention. The suburothelial protocol brought intermediate results, worse than the detrusor but better than the trigone-only technique. A small placebo-controlled trial with bladder pain syndrome (BPS) patients comparing OnabotA 100 U or saline, injected at the trigone, showed the superiority of the toxin in pain relief and voiding frequency [34]. Vesico-ureteral reflux was never reported after BoNT/A trigonal injection [35,36,37]. There is no evidence, however, that trigonal injections will increase the persistence of OABi patients in treatment.

Another variation investigated was the volume of the BoNT/A solution injected into each site. Instead of the approved 1.0 mL or 0.50 mL per injection site, volumes of 0.25 mL [38], 0.20 mL [39], or even 0.10 mL [40] were assessed. Since comparative studies were not carried out, it is impossible to confirm that lower volumes at each injection site make treatment less painful. The small volumes injected per site may, however, reduce efficacy due to the large amount of toxin that can be lost by backflow of the injected liquid once the needle is removed. In addition, larger volumes may facilitate the diffusion of the toxin. An animal study demonstrated that the amount of cleaved SNAP-25 induced in the bladder wall by a fixed dose of OnabotA was directly related to the injection volume [41].

The reduction in the number of injection sites was investigated to speed up the BoNT/A administration and decrease pain [18]. A study with NDOi patients that reduced the number of injections to 10 reported that the procedure was quicker and less painful, while providing an efficacy equivalent to the approved protocol at 24 weeks follow-up [42]. Interestingly, such protocol permitted the distribution of the fluid throughout 1/3 to 1/4 of the detrusor layer, as determined by magnetic resonance imaging [42]. A study with NDOi patients treated with AbobotA 750 U, concluded that the delivery of the toxin by 15 or 30 intra-detrusor points provided similar results [43].

More extreme reductions of the injection points, to four or even fewer, were investigated. One or three injection sites in the posterior bladder wall were used in a small cohort of NDOi and OABi patients to deliver OnabotA 100–300U [44]. ICIQ-SF score improvement of more than 5 points was achieved in 55% of OABi and 91% of NDOi patients. The overall subjective success rate was 69%. In another mixed NDOi and OABi cohort, 3–4 injections of 2 mL each at the equatorial line of the bladder were used to deliver OnabotA 100–300 U [45]. Administration was easy to make under local anesthesia, and clinical efficacy was good, with 81% of patients achieving continence. The duration of the effect was 34.9 weeks, similar to that observed in the approved protocol. Although reducing injection points could, in theory, decrease the incidence of post-treatment UTIs, these last two studies did not unequivocally confirm this. The incidence of UTIs was 11.1% after 1–3 [44] and 24% after 3–4 injections [45]. Long-term adherence to these protocols was not reported.

As attractive as it might be, no randomized clinical trials demonstrate that protocol simplifications increase patient adherence to BoNT/A treatment. Moreover, all require the use of cystoscopy and maintain a significant risk of UTIs, and cannot exclude the risk of urinary retention. Yet, the discomfort associated with a low volume per injection point or a low number of injection points must be compared to the discomfort associated with the classical method. In addition, it must be investigated if the simplified protocols confer the same efficacy and duration of effect while reducing the rate of UTIs and decreasing the risk of urinary retention.

In conclusion, if adherence to urinary incontinence treatment with BoNT/A is to be increased, particularly among OABi patients, other forms of toxin administration that do not require cystoscopy and bladder wall injections must be investigated. The present advances in this field will be discussed in the next section.

## 4. Transporters or Facilitators for the Passage of the Toxin Across the Urothelium to Avoid Cystoscopy

An alternative to bladder injections oriented by cystoscopy to deliver BoNT/A may not be an easy task to discover, as the large free form of the BoNT/A toxin molecule does not cross the bladder urothelium. In addition, the molecule has an inherent structural instability leading to quick degradation. Animal studies showed that the simple instillation of a high dose of OnabotA dissolved in saline did not cleave SNAP-25 in nerve fibers coursing the bladder wall [41]. Therefore, several methods were investigated to overcome this problem.

The Electromotive Drug Administration (EMDA) refers to the use of a low-intensity electrical current to move drugs across issues. In the case of the bladder, an indwelling catheter is connected to the anode component of the current generator, while the skin electrodes placed over the anterior abdominal wall are connected to the cathode component. The bladder is filled with a solution containing a cationic drug, the penetration of which across the bladder wall is enhanced when the electric current is applied. EMDA equipment is commercially available (ex: Medolla, MO, Italy) and authority-approved for multiple applications (skin, bladder, sclera) [46].

The passage of the free toxin across the urothelium using EMDA has been demonstrated in experimental conditions. A study in rabbits identified AbobotA by immunostaining with well-spread staining throughout the muscular layer of the bladder after intravesical EMDA administration [46]. Positive AbobotA staining was also identified in the pelvic nerve, sacral nerve plexus, spinal cord, intestinal wall, and pelvic floor muscles. The study, however, did not provide information concerning the extension of cleaved SNAP-25 in nerve fibers coursing through the bladder wall [46].

According to the studies available, a maximal current of 100 mA (10 mA increment/s) is applied for about 20 min to avoid thermal damage to tissues. [47]. For small molecules, the penetration across the human urothelium is limited to the mucosa. Thus, it is unclear if a large, fragile molecule such as BoNT/A can reach the muscular layer in its intact, functional form. That is probably the reason why EMDA has been mainly investigated in children who have thinner bladder walls. Most of the information derives from one single center that repeatedly reported good results using a solution containing 10 U/kg of AbobotA. In a long-term follow-up, which summarizes the experience with 24 children or young adolescents with myelomeningocele, 18 (75%), 11 (45.5%), 9 (37.5%), 8 (33%), and 7 (29.1%) were completely dry between clean intermittent catheterizations at 1, 2, 3, 5, and 6 years of follow-up, respectively. A decrease in maximum detrusor pressure and an increase in maximal cystometric capacity were reported. Skin erythema and burning sensation seem to be the only side effects related to the EMDA procedure [48]. These results could not be reproduced by Koh and co-workers in a similar pediatric population. Twelve children received 14 treatments using EMDA to deliver OnabotA or AbobotA. No statistically significant improvements in maximal cytometric capacity and maximal detrusor pressure were demonstrated. Eight patients subsequently received conventional intravesical BoNT/A injections with significant improvements in both urodynamic parameters and in symptoms [49]. A recent work using EMDA to deliver OnabotA 100 U in 43 adults with OAB was presented recently in abstract form [50]. Urinary frequency, urgency, and maximal bladder capacity showed a remarkable improvement from baseline at 3-, 6-, and 12-months post-treatment. No information was provided regarding the eventual effect of OnabotA administration by EMDA on urgency urinary incontinence. However, the fact that sensory mechanisms play an important role in OABi, EMDA, by potentially enhancing the passage of BoNT/A until the mucosa, may be relevant to reach sensory fibers. No serious adverse events were observed during BoNT/A administration by EMDA. Nevertheless, the efficacy of the method needs further investigation, preferably by large randomized clinical trials, before being accepted as an alternative method to the classical bladder injections of the toxin in NDO and OABi patients.

Low-energy shock waves (ESWL) have been used to facilitate the application of BONT/A to skeletal muscles. This technique was recently investigated to tear the urothelium barrier and facilitate the passage of BoNT/A instilled into the bladder to the bladder wall. In 15 OABi patients, OnabotA 100 U was instilled in a volume of saline equivalent to half the bladder capacity. A total of 3000 shocks were then delivered for 10 min in the supra-pubic area. Symptomatic improvement was captured by the OAB Symptom Score, which lasted only 2 months [51]. In an experimental study in rats, low-energy shock waves were applied to the bladder filled with a BoNT/A solution. The method decreased bladder inflammatory response to chemical injury and marginally decreased the expression of the intact form of SNAP-25 compared with the control group. The expression of cleaved SNAP-25 was not investigated [52]. Despite this encouraging evidence, no further studies were carried out. At this moment, more studies are necessary to confirm the utility of ESWL to apply BoNT/A instilled in the bladder and avoid wall injections.

OnabotA 300 U dissolved in 50 mL of 50% solution of dimethyl sulfoxide (DMSO) improved incontinence in 25 OABi women at 1 but not at 3 months, despite the improvement of urgency at both time points [53]. The study was not controlled, leaving unclear whether the observed effect was due to the toxin or to the DMSO, which easily crosses cellular membranes. Although no serious adverse effects were detected, this method of administration did not see further developments.

Bladder instillation of OnabotA encapsulated in liposomes was shown to cross the rat urothelial barrier, as revealed by the appearance of cleaved SNAP-25 in bladder nerve terminals [54]. However, this experimental finding did not demonstrate efficacy in pilot studies. OABi patients were randomized to intravesical instillation of 200 U of BoNT/A encapsulated in 80 mg of liposomes, dissolved in saline. At 1 month, the liposome group showed very mild improvements in urinary frequency and in urgency episodes [55]. In a similar study with BPS patients, liposomal-formulated OnabotA 200 U did not show any superiority over oral pentosan polysulfate sodium [54]. Although no adverse events were reported, the available clinical evidence is not encouraging to replace cystoscopy-guided bladder injections with a simple instillation of BoNT/A encapsulated in liposomes.

A singular thermal-reversal hydrogel (TC-3), capable of forming a gelatinous clot that adheres to the urothelium at body temperature, was investigated as a method for storing the toxin and promoting its prolonged release in the bladder. In a single-arm study, the hydrogel containing BoNT/A was safe but did not bring relevant symptomatic improvement to BPS patients [56]. In another study, 39 patients with OABi were randomized into four small groups: a control (n = 11) and three active-treated groups (TC-3 gel plus 200 U OnabotA, n = 9; TC-3 gel plus 200 U OnabotA plus DMSO, n = 10; and DMSO only, n = 9). No robust changes were observed within each of the four groups. Although a trend in favor of TC-3 gel plus 200 U OnabotA was suggested, no further developments exploring the thermal hydrogel were pursued in larger cohorts [57]. Table 2 summarizes the above mentioned studies. 

Nano-formulations to encapsulate BoNT/A represent a noticeable breakthrough in the delivery of the toxin. Thomas Harris demonstrated a new strategy for the delivery of BoNT/A through the use of a Polyelectrolyte Complex (PEC) system to encapsulate the active toxin in polymeric nanoparticles. The release kinetics of the nanoparticle-based formulations exhibited a stable, linear release profile over time. Within 30 days, approximately one-third of the encapsulated protein was released, and by 3 months, the cumulative release reached three-quarters of the total encapsulated toxin [58]. Nano-formulations of OnabotA have high stability to heat and to pH changes and are more resistant to enzymatic degradation, including that induced by bacterial enzymes, a characteristic that may be of great value when the administration is carried out in the bladder of patients voiding by intermittent catheterization (see [59]. Nanoparticles also have an ideal size for cellular endocytosis [59]. BoNT/A nano-emulsions were tested in mice and subsequently in patients with severe forehead wrinkles. Complete paralysis of the forehead muscles occurred after the application of BoNT/A nano-emulsion cream, as evidenced by the subject’s inability to elevate the brow, which persisted for up to 12 weeks. The therapeutic efficacy of the polyelectrolyte complex was also confirmed by quantitative forelimb grip strength assay in animal models [58]. The silver colloidal nanoparticles were another recently patented nano-formulation that stabilizes the toxin transportation and protects BoNT/A from degradation [60]. In a pilot small clinical trial, muscular injections of BoNT/A in colloidal silver particles substantially prolonged paralysis compared to control patients who received the conventional toxin formulation. Effects lasted up to 8.5 months, thus doubling the 3–4 months encountered with conventional formulas, and no significant side effects were detected. Whether these new formulations will also operate in the bladder and help BoNT/A to cross the urothelium and reach parasympathetic and sensory neurons in the different layers of the bladder has not yet been investigated. Even if injections are necessary, it is exciting to envisage that nanoparticles could prolong the effect of the toxin injected into the bladder wall and therefore decrease the frequency of cystoscopies needed in clinical practice.

In summary, at this moment, the only innovations that are worth testing in large clinical trials are EMDA and ESWL, although the costs of such trials will be more dependent on national authorities than on industry due to the cost involved and the revenue expected. All the others either did not succeed in pilot studies (DMSO as solvent, BoNT/A encapsulation in liposomes, or combination with thermal reversal hydrogels) or were not tested experimentally or clinically in the urinary bladder, as is the case of BoNT/A encapsulated in nanoparticles.

## 5. Ultrasound-Guided Transabdominal Botulinum Toxin Injection of the Bladder

To avoid the necessity of cystoscopy to administer BoNT/A in the bladder wall, a recent study compared the injection of BoNT/A 100 U performed under real-time ultrasound guidance through the abdominal wall with the standard transurethral cystoscopic injections, in 64 OABi patients randomized 1:1 [61]. In the ultrasound-guided protocol, the toxin was divided into four injections of 2 mL each, administered in the anterior bladder wall (left, right, top, and bottom). Both groups showed improvements in urgency episodes, daytime frequency, nocturia episodes, and bladder capacity, without differences between the groups at 1 and 6 months after the toxin administration. However, patients treated transabdominally reported less pain, had a lower incidence of hematuria and UTIs, and a higher willingness to continue the treatment. The reproducibility and efficacy of ultrasound-guided injections need to be investigated in large multicenter randomized clinical trials, and in posterior real-world studies to determine patients’ adherence to the method.

## 6. What Can Be Done in the Short-Term to Improve Patients’ Adherence to BoNT/A While New Methods for Bladder Administration Are Under Testing?

The literature is not yet conclusive in terms of which barriers have which role in the NDOi and OABi population in real-world conditions. The relative role of various barriers to long-term adherence may vary from patient to patient, and predictors of adherence should be identified to tailor individual management. Overactive bladder (OAB) symptoms increase with age and can be associated with multiple comorbidities. Intravesical BoNT-A injection is effective for OABi frail patients. However, male sex, high post-void residual urine, and the presence of comorbidities are independent risk factors for adverse events such as UTIs and urinary retention. A review showed that after BoNT/A treatment, 60% of frail elderly patients had a post-void residual volume > 150 mL, and 11% had urinary retention. While BoNT-A injection did not cause significant adverse events in Parkinson’s disease patients, those with cerebrovascular accidents had more voiding difficulties, and diabetic patients were at increased risk of urinary retention [62]. In NDOi patients, a low bladder compliance should be a reason to refuse BoNT/A treatment. Adherence will be low, and the upper urinary tract will remain at risk of deterioration. Therefore, careful patient selection and thorough explanation of the potential risks of BoNT/A bladder injection should be given before the procedure.

If undoubtedly a close interaction between patients and caregivers increases adherence [29], institutions should create a mechanism for supporting patients, which might permit the detection of an early failure, the diagnosis of adverse events, an effective combat against fatigue related to the chronic treatments, and to book new treatments.

The combination of BoNT/A with anticholinergic drugs or β3 agonists was never investigated in large randomized clinical trials. However, it may be a practice that might contribute to decreasing the frequency of intravesical BoNT/A injections. In long-term OAB trials with OnabotA 100 U [15], patients enrolled were prohibited from using anticholinergics. So, comparison of the OnabotA effect in anticholinergic users and non-users is not possible. Nevertheless, it is conceivable that outside clinical trials, many physicians and OAB patients will try to prolong the tail effect of OnabotA 100 U injections with anti-cholinergic compounds or β3 agonists. This practice might explain the long intervals between treatments seen in some real-world cohorts [27,30]. In patients with NDOi [63], the improvement in the number of episodes of urinary incontinence obtained with OnabotA 200 U was similar among anticholinergic user and non-user patients. However, it remains to be determined whether patients who responded partially to an anticholinergic may receive additional benefit from combination therapy.

## 7. Future Directions and Concluding Remarks

Several of the above approaches will involve not only the drug to be delivered, BoNT/A, but also devices that serve as delivery tools. Regulatory agencies consider the combination of a drug and a device as a “combination product” (CP). Following the US Safe Medical Devices Act of 1990, regulatory agencies, including the US Food and Drug Administration, the European Medicines Agency, and the Korean Ministry of Food and Drug Safety, have developed and/or updated their guidelines [64]. While these guidelines share similarities, they also differ in other aspects. Given that the development of CPs can be costly, it is likely to need the involvement of pharmaceutical and/or device companies. To get a CP product approved, it will need to follow the applicable guidelines in the jurisdictions where it is to be commercialized. While some developments coming out of academia may not need such regulatory approval, this would imply that they remain off-label treatments, which in many countries has implications on liability of the physicians administering it and on reimbursement.

Large pivotal trials have standardized the technical aspects of the injection procedure. However, the necessity of cystoscopy is a serious drawback that may be behind the low adherence to long-term treatment with BoNT/A, particularly among MS and OABi patients. A decrease in the number of injections used in the pivotal studies may facilitate the procedure, but does not eliminate the need for cystoscopies to inject the toxin into the bladder wall. Moreover, cystoscopy is a potential cause of the high number of UTIs observed with the present approved technique. Transabdominal delivery of BoNT/A to the bladder wall is attractive, but its reproducibility is unclear. The new formulations of the BoNT/A molecule that may facilitate its passage across the urothelium are under intense investigation. However, human studies are necessary before they can replace the present method of bladder BoNT/A administration. In addition, it is not foreseeable at the moment which devices will need to be developed to deliver new BoNT/A formulations. As none of the innovative approaches to BoNT/A delivery has yielded robust data until now, it remains speculative which of these will merit entering clinical practice. Where applicable, this may require adherence to regulatory guidelines for the development of CPs. Given the investment required to fulfill regulatory requirements, i.e., complete phase III studies, decisions by pharmaceutical companies may not only be driven by medical needs but also by economic considerations, i.e., whether such investment is likely to yield adequate returns.

From a purely medical perspective, the community will need to find a balance between procedure costs, repeat visits, equipment needs for delivery tools, and scalability of the manufacturing process for carriers. A realistic assessment of the net benefit to patients will only be possible once we have more robust data on the efficacy and tolerability of each novel treatment.

## Figures and Tables

**Table 2:** Summary of studies assessing new delivery modalities of BONT/A in the bladder. W—weeks, M—months, Y—years, U—units.

Modalities	Study Type	Duration	Patients/Animals	Dose	Outcome	Ref.
Intradetrusor injections (1–3 points)	Non-controlled pilot study	31 w	45 adults with OAB or NDO refractory/intolerant to medical treatment	100–300 U OnabotA	ICIQ-SF improvement similar efficacy/adverse events to standard	[36,44]
Intradetrusor injection (3–4 points)	Non-controlled pilot study	35 w	21 OAB/NDO previously responsive to standard multi-site BoNT-A	100–300 U OnabotA	86% improved (TBS) 81% continent duration similar to prior standard template UTI 24%	[45]
Intravesical installation of BoNT/A dissolved in dimethyl sulfoxide	Phase 1/2 non-controlled clinical study	1 and 3M	25 women with OAB	OnabotA 300 U dissolved in 50 mL of 50% DMSO	Safe and feasible Reduction in urgency episodes and improved bladder capacity only at 1 month. Mild adverse effects	[53]
Intravesical electromotive BoNT/A administration	Experimental study (rabbit)	1 M	5 BoNT/A EMDA 5 controls	10 IU/Kg OnabotA	BoNT/A immunostaining was demonstrated in the bladder and bowel biopsies	[41]
	Clinical study	6 W	12 children with NDOi	100 U OnabotA	No changes in maximal cystometric capacity and maximal detrusor pressure	[49]
	Long-term follow-up clinical study	6 Y	44 children with NDOi		Sustained improvement in continence and bladder compliance Repeat treatments feasible	[48]
	Single center clinical study	12 M	54 adults with OABi		Urgency and frequency improvement Good safety profile Repeat EMDA feasible	[50]
Intravesical BoNT/A delivered with low-energy shockwaves	Experimental study (rat)	24 h	12 adult rats	100 U OnabotA	Increased bladder capacity and reduced detrusor overactivity after acetic acid Decreased expression (trend) of SNAP-25 by immunostaining	[52]
	Clinical study	1 M	15 adults with OAB		Feasible and safe Improvement in urgency and frequency No major adverse events	[51]
Liposome-encapsulated onabotulinumtoxinA (intravesical instillation)	Experimental study in rats	8 days	Adult female rats	BoNT/A 20 U/kg (saline or encapsulated in liposomes)	Less detrusor contractions after acetic acid instillation and less SNAP-25 expression after treatment BoNT/A encapsulated in liposomes	[54]
	Single-center, placebo-controlled study	1 M	24 adults with OAB	200 U OnabotA	Small albeit significant improvement in urgency episodes and OAB symptom scores; no serious adverse events	[55]
	Prospective, controlled clinical study	8 w	24 patients with IC/BPS randomized 1:1	Intravesical OnabotA-liposome (80 mg/40 cc) or oral pentosan polysulfate sodium (100 mg)	No difference between the groups in the clinical parameters. Liposome instillation did not cause urinary incontinence, retention, or infection. No adverse events or symptoms worsening	[54]
Intravesical instillation of BoNT/A in TC-3 hydrogel	Prospective, open-label pilot study	1 M	12 adults IC/BPS	200 U OnabotA	Feasible and safe Improvement in pain and symptom scores over 1 month. No adverse events	[56]
	Double-blind randomized pilot study with 4 arms	1 M	39 females with OAB	Active arm 200 U OnabotA in TC-3 hydrogel	No statistically significant changes were observed within the group	[57]

## Data Availability

No new data were created or analyzed in this study.
